# Enhanced myelopoiesis and aggravated arthritis in *S100a8*-deficient mice

**DOI:** 10.1371/journal.pone.0221528

**Published:** 2019-08-22

**Authors:** Annabelle Cesaro, Joan Defrêne, Asmaa Lachhab, Nathalie Pagé, Mélanie R. Tardif, Amin Al-Shami, Tamas Oravecz, Paul R. Fortin, Jean-François Daudelin, Nathalie Labrecque, Fawzi Aoudjit, Martin Pelletier, Philippe A. Tessier

**Affiliations:** 1 Axe de recherche sur les maladies infectieuses et immunitaires, Centre de recherche du CHU de Québec-Université Laval, Quebec city, Quebec, Canada; 2 Lexicon Pharmaceuticals, Inc., The Woodlands, Texas, United States of America; 3 Département de médecine, Faculté de Médecine, Université Laval, Quebec City, Quebec, Canada; 4 Maisonneuve-Rosemont Hospital Research Centre, Montreal, Quebec, Canada; 5 Department of Medicine and Department of Microbiology, Infectiology and Immunology, Université de Montréal, Montréal, Canada; 6 Département de microbiologie-infectiologie et d’immunologie, Faculté de Médecine, Université Laval, Quebec City, Quebec, Canada; University of Roehampton - Whitelands College, UNITED KINGDOM

## Abstract

Expressed strongly by myeloid cells, damage-associated molecular pattern (DAMP) proteins S100A8 and S100A9 are found in the serum of patients with infectious and autoimmune diseases. Compared to S100A9, the role of S100A8 is controversial. We investigated its biological activity in collagen-induced arthritis using the first known viable and fertile *S100a8*-deficient (*S100a8*^*-/-*^) mouse. Although comparable to the wild type (WT) in terms of lymphocyte distribution in blood and in the primary and secondary lymphoid organs, *S100a8*^*-/-*^ mice had increased numbers of neutrophils, monocytes and dendritic cells in the blood and bone marrow, and these all expressed myeloid markers such as CD11b, Ly6G and CD86 more strongly. Granulocyte-macrophage common precursors were increased in *S100a8*^*-/-*^ bone marrow and yielded greater numbers of macrophages and dendritic cells in culture. The animals also developed more severe arthritic disease leading to aggravated osteoclast activity and bone destruction. These findings were correlated with increased inflammatory cell infiltration and cytokine secretion in the paws. This study suggests that S100A8 is an anti-inflammatory DAMP that regulates myeloid cell differentiation, thereby mitigating the development of experimental arthritis.

## Introduction

Analogous to pathogen-associated molecular patterns, damage-associated molecular patterns or DAMPs, also known as alarmins, are endogenous molecules released passively by cells undergoing non-programmed cell death as well as actively through normal secretion pathways [1]. They are believed to play key roles in the progression of inflammatory diseases such as rheumatoid arthritis [2], systemic lupus erythematosus [3] and inflammatory bowel disease [4]. The DAMPs S100A8 and S100A9 belong to a subset of S100 proteins called myeloid related proteins (MRPs) because they are predominantly expressed in neutrophils and monocytes [5]. These include S100A8 and S100A9, which are expressed constitutively in myeloid cells and are inducible in synoviocytes[6], keratinocytes[7], epithelial cells [8], endothelial cells [9] and other cell types. S100A8 and S100A9 form non-covalently bonded homodimers and a heterodimer called S100A8/A9 or calprotectin [10]. The three dimers are not always co-expressed [9] and are secreted independently during inflammatory responses through alternative secretion pathways independent of Golgi and secretion vesicles [11, 12]. It is therefore presumed that they have different activities.

While S100A9 has been studied extensively, the activities of S100A8 remain controversial. S100A9 stimulates pro-inflammatory cytokine secretion [13, 14], neutrophil phagocytosis [15], degranulation of secretory and specific/gelatinase granules [16] and phagocyte migration [13, 17] and promotes the differentiation of acute myeloid leukemia cells [18]. *S100a9*^*-/-*^ mice have been found resistant to adjuvant-induced arthritis and systemic lupus erythematosus, the latter resistance being due at least in part to reduced CD8^+^ T cell activation [19, 20]. Thus, studies using these mice have shown the importance of S100A9 as an alarmin in immune cell crosstalk and in the establishment of persistent inflammation. S100A8 is found at heightened levels in chronic inflammation, but its role therein remains uncertain. S100A8 is a chemotactic factor for neutrophils and monocytes [21], and injecting it into live animals leads to accumulation of phagocytes at the inflammatory site [22, 23]. Inhibiting it with antibodies reduces leukocyte recruitment in models of acute inflammation [24, 25], which is consistent with pro-inflammatory activity. However, its expression by macrophages is induced by glucocorticoids and IL-10 [26, 27], and S100A8 itself induces IL-10 expression [28], suggesting an anti-inflammatory function. S100A8 is easily oxidized [29–32] and provides some protection against the harmful effects of reactive oxygen species (ROS) released in chronic inflammation [33]. Oxidized S100A8 is anti-inflammatory [34] and reduces IgE-mediated mast cell degranulation and cytokine secretion [33]. It is thus safe to say that both pro-inflammatory and anti-inflammatory activities have been reported for S100A8.

S100A8 has been shown to promote the commitment of hematopoietic stem cells and progenitor cells to myelogenesis in experimental arthritis [18, 35]. Its expression is increased in hematopoietic stem cells and early progenitors in arthritic mice, and treating Kit^+^Sca1^+^Lin^-^ cells from such mice with recombinant S100A8 increases production of Gr1^+^CD11b^+^ cells and osteoclasts. However, S100A8 inhibits the differentiation of acute myeloid leukemia cells into mature neutrophils and monocytes [18, 35]. As is the case for inflammation, the exact role of S100A8 in the production and maturation of myeloid cells remains unknown.

Levels of S100A8/A9 in the serum and synovium of rheumatoid arthritis patients have been found to correlate with disease severity [36]. Both proteins are also expressed constitutively in bone and cartilage cells [37]. While blocking S100A9 prevents inflammation and joint destruction by reducing leukocyte migration as well as cytokine secretion [6, 13, 38], the functions of S100A8 in chronic inflammation are unknown, in part due to the lethality of the deleted *S100a8* gene in embryonic mice [39, 40]. In this study, we characterized the first viable *S100a8* knockout mouse strain and investigated the role of S100A8 in the differentiation of myeloid cells. We report that its absence is strongly associated with increased numbers of circulating myeloid cells and aggravated collagen-induced arthritis, suggesting that it has a mitigating effect on the immune response in arthritis. We therefore propose that DAMPs can exert inhibitory activities in inflammatory diseases.

## Materials and methods

### Generation of S100a8-deficient mice

The animal protection committee at Université Laval (Quebec City, QC, Canada) approved the experimental protocols (approval numbers 2012103 and 2013006). The *S100a8* targeting vector was derived using the Lambda KOS system [41]. The Lambda KOS phage library was screened by PCR using primers specific for exons 2 and 3 (Table 1). The PCR-positive phage super-pools were plated and screened by filter hybridization. Three pKOS genomic clones, pKOS-18, pKOS-23 and pKOS-64, were isolated from the screened library and confirmed by sequence and restriction analysis. Gene-specific arms 5’-GCGACTTTTCCTTTCAGTTGAAAGGAAATCTTTCGTGACA-3’ and 5’-AGGACCAAAACAAGACAGTTCTTTCCAGTTTTTCATCCC-3’ were appended by PCR to a yeast selection cassette containing the URA3 marker. The yeast selection cassette and pKOS-18 were co-transformed into yeast, and clones that had undergone homologous recombination to replace a 529 bp region containing exons 2 and 3 with the yeast selection cassette were isolated. The yeast cassette was subsequently replaced with a LacZ/Neo selection cassette to complete the *S100a8* targeting vector. The NotI linearized targeting vector was electroporated into 129/SvEvBrd (Lex-1) ES cells. G418/FIAU-resistant ES cell clones were isolated and correctly targeted clones were identified and confirmed by Southern blot analysis (data not shown). Three targeted ES cell clones were identified and microinjected into C57BL/6 (albino) blastocysts to generate chimeric animals that were bred to C57BL/6 (albino) females, and the resulting heterozygous offspring were interbred to produce homozygous *S100a8*-deficient mice. The genotype at the *S100a8* locus was determined by screening DNA from tail biopsies using quantitative PCR for the Neo cassette. This strategy allowed discrimination of 0, 1, or 2 gene disruptions representing respectively *S100a8*^*+/+*^, *S100a8*^*+/-*^ and *S100a8*^*-/-*^ mice.

**Table 1:** PCR primers used for *S100a8*^*-/-*^ mice generation and mRNA study.

	Primers	
Targeted gene	Foward	Reverse
*S100a8* in Lambda KOS phage library	a8-1 [5’-GAAATCTTTCGTGACAATGCCG-3’]	a8-7 [5’-GGAACTCCTCGAAGTTAATTG-3’]
5’ internal probe 32/33	a8-32 [5’-AGGGGCCTAGACATGGACTTATTG-3’]	a8-33 [5’-TACGCCATTCCACTTCCTTTATCC-3’]
3’ external probe 40/41	a8-40 [5’-GCTTTGCCTTTTGTGGAGATC-3’]	a8-41 [ 5’-GGTTGTAAACTACATTCCCAG-3’]
*S100a8*^*+/+*^	*S100a8*-46 [5’-CAGGGGCCTAGACATGGACTTAT-3’]	*S100a8*-4 [5’ GTGGTAGACATCAATGAGGTTG-3’]
*S100a8*^*-/-*^	*S100a8*-46 [5’CAGGGGCCTAGACATGGACTTAT-3’]	GT-IRES [5’- GCTAGACTAGTCTAGCTAGAGCGG-3’]
*S100a9*	*S100a9*-F [5’-GCCAACAACCTGTGTAGTACTGTGC-3’]	*S100a9*-R [5’-GGGTGAAATAGAGGAGGCTGTTAGG-3’]
S100A8 cDNA	S100A8-F [5'-GGAAATCACCATGCCCTCTA-3']	S100A8-R [5'-TGGCTGTCTTTGTGAGATGC-3']
S100A9 cDNA	S100A9-F [5'-TCATCGACACCTTCCATCAA-3']	S100A9-R [5'-GTCCTGGTTTGTGTCCAGGT-3']
18S cDNA	18S-F [5’-TGCAAGCTTATGACCTGCAC- 3’]	18S-R [5’-CAAGTGGCGTTGAGCAATAA-3’]

*S100a8*^*-/-*^ mice (mixed 129/SvEvBrd and C57BL/6J genetic background) were backcrossed to DBA/1 and C57BL/6 backgrounds (Jackson Laboratories) for 10 generations. Routine genotyping for screening was carried out by PCR with tail biopsy samples (Table 1).

### Expression analysis of S100a8 and S100a9 by RT-PCR

Total RNA was extracted from bone marrow cells with Trizol, followed by reverse transcription (RT) carried out according to the manufacturer’s instructions (Promega). PCR amplifications of *S100a8*, *S100a9* and *18S* cDNA were performed using primers described in Table 1.

### Protein and antibody production

Recombinant murine S100A8 (mS100A8), rat monoclonal anti-mS100A9 (clone 2A5) and rabbit polyclonal anti-mS100A8 and anti-mS100A9 IgG were generated as described previously [13, 22, 23]. The absence of endotoxin contamination in antibody and protein preparations was confirmed using the Limulus amebocyte assay (Cambrex).

### Flow cytometry

Mice were sacrificed at the age of 6–8 weeks by cervical dislocation, and the blood and femurs were recovered. Bone marrow cells were collected from the femurs by flushing with RPMI medium supplemented with 5% foetal calf serum. Erythrocytes were lysed by suspending the cells in 0.15 M NH_4_Cl for 5 min followed quickly by centrifugation. Leukocytes were stained with Live/Dead Fixable Blue Dead Cell Stain (Invitrogen, Paisley, UK) and Fc receptors on cells were blocked by incubation with Fc block (eBioscience/Thermo Fisher, San Diego, CA, USA). Cell labelling was performed using a combination of monoclonal antibodies (S1 Table). Intracellular antigen was detected inside permeabilized cells using the FoxP3/Transcription factor staining buffer set (eBioscience) and labelling with monoclonal antibodies. Cell fluorescence was then analyzed on a BD LSR/LSRII (BD Biosciences, Mississauga, ON, CA) and data was analyzed using the FlowJo 10 (Tree Star Inc.) and Cytobank software (Cytobank, Santa Clara, CA). The viSNE was run using default Cytobank parameters (iterations = 1000, perplexity = 30, θ = 0.5). The number of live cells analyzed ranged from 30,000 to 100,000 per sample. Samples were down-sampled randomly and analysis was run on equal numbers of events per sample. The range of events was determined from the sample with the fewest events acquired. The viSNE heat maps are shown as fluorescent intensity of each marker for each event.

### Acute inflammation models

Air pouches were raised on the dorsum of 10–12-week-old mice by subcutaneous injection of 3 mL of sterile air on days 0 and 3 [22]. On day 7, 1 mL of LPS (1 μg/mL) or its diluent (PBS) was injected into the air pouches. Six hours after injection, the mice were sacrificed by asphyxiation with CO_2_. The air pouches were washed with 5 mL of PBS-5 mM EDTA and the exudates were centrifuged at 500 × g for 5 min at 4°C. For thioglycollate-elicitation, 1 mL of 4% Brewer's thioglycolate broth (Becton Dickinson, Franklin Lakes, NJ, USA) was injected into the abdomen. The mice were euthanized 4 h later and peritoneal cells were recovered by lavage with 5 mL of PBS-5 mM EDTA. Cells were counted with a hematocytometer following acetic blue staining. Leukocyte subpopulations were characterized on the basis of Wright-Giemsa staining of cytospins.

### Isolation and culture of bone marrow cells

Bone-marrow-derived dendritic cells and macrophages were prepared as described previously [42, 43]. Briefly, bone marrow cells were isolated from femurs and cultured in RPMI 1640 medium supplemented with 10% heat-inactivated foetal bovine serum, 1X non-essential amino acids, 50 μM β-mercaptoethanol and 0.2% primocin. The dendritic cells were cultured in the presence of murine GM-CSF (15 ng/mL) and murine IL-4 (15 ng/mL). On day 6, non-adherent cells were collected and cultured for a further 48 h in the presence of 50 ng/mL each of GM-CSF and IL-4. Non-adherent cells were then harvested and cultured for 24 h in the presence or not of LPS (300 ng/mL). Macrophages were cultured in the presence of GM-CSF (10 ng/mL) for 4 days, and non-adherent cells were then harvested and cultured in fresh media for an additional 3 days. Adherent cells were detached with trypsin and harvested for analysis at day 7.

### Mixed lymphocyte reaction

CD4^+^ T cells were purified from the spleens of B10.BR mice (Jackson Laboratory) using the EasySep™ Mouse CD4^+^ T Cell Isolation Kit (STEMCELL Technologies) following the manufacturer’s instructions. Cells were then labelled with 5 μM CFSE (carboxyfluorescein succinimidyl ester) according to the manufacturer’s instructions (Invitrogen) before being co-cultured with bone-marrow-derived dendritic cells at different T-cell/dendritic cell ratios (1:5, 1:10, 1:20) for 72 h in RPMI 1640 medium supplemented with 10% heat-inactivated foetal bovine serum, 50 μM β-mercaptoethanol and 0.2% primocin. Cells were then harvested and lymphocytes were labelled for flow cytometry analysis.

### ROS and phagocytosis assays

Neutrophils were purified from bone marrow using the Neutrophil Isolation Kit according to the manufacturer’s instructions (Miltenyi, Bergisch Gladbach, Germany) then exposed to 1 μM CellROX™ reagent (Invitrogen) and diphenyleneiodonium (DPI; 5 μM) and/or rotenone/antimycin A (0.5 μM) for 30 minutes followed by PMA (100 nM) for 1 h. Cells were then washed with PBS and analyzed by flow cytometry. Phagocytosis was measured using the Vybrant™ Phagocytosis Assay Kit according to the manufacturer’s instructions (Invitrogen). The cells were incubated for 2 h at 37°C with particles diluted 1:3 in RPMI medium supplemented with 10% foetal calf serum then washed with PBS and analyzed by flow cytometry.

### Oxygen consumption

Bone marrow neutrophils were seeded in XF96 culture plates (3x10^5^ cells/well) in XF media (Seahorse Bioscience/Agilent Technologies). The plate was centrifuged for 1 min at 600 rpm to allow adherence of neutrophils. PMA (10^−7^ M) and DPI (5 μM) were injected at the time points indicated in the figures. Measurements were made in real-time in the extracellular flux analyzer as described previously [44]. The Wave 2.3 software provided automatic calculation of the oxygen consumption rate.

### Osteoclast culture and resorption assay

Bone marrow cells were cultured for 7 days in RPMI + 10% foetal bovine serum + 30 ng/mL rmM-CSF + 30 ng/mL rmRANK-L + 0.2% primocin at 37°C in osteoassay plates (Corning). The medium was then acidified with HCl to maintain the pH at 7.1 for 3 days. The cells were then lysed with 10% NaClO for 5 min and washed 2 times with distilled water. Resorption pits were counted and measured by microscopy.

### Induction of collagen-induced arthritis

Female mice (WT and *S100a8*^*-/-*^ DBA/1) aged 6–8 weeks were immunized subcutaneously at the base of the tail with chicken collagen type II (100 μg/mouse, Chondrex Inc.) emulsified in complete Freund’s adjuvant then 26 days later received an intra-peritoneal injection of LPS (25 μg/mouse). Disease activity was monitored every other day on a scale of 0 to 4 per paw for a maximum score of 16 per mouse as described previously [13]. For antibody treatment, mice received rabbit polyclonal anti-S100A8 or control IgG three times per week (10 mg/kg of body weight, intra-peritoneal) starting on the day of LPS injection.

### Histopathological assessment of collagen-induced arthritis

Paws were fixed in 4% paraformaldehyde, decalcified in Surgipath (Leica Biosystems) for 10 days and embedded in paraffin. Sections were stained with H&E (Thermo Fisher Scientific) or safranin/fast green colorations (VWR International). Bone destruction, collagen integrity and cell infiltration were evaluated by two impartial observers as described previously [13] according to the following scales: 0–3 for bone destruction; 0–2 for collagen; 0–2 for cell infiltration.

### Micro-Computed Tomography (μCT) analysis

Paws fixed in 10% formalin were scanned in a μCT device (SkyScan) at the McGill University Centre for Bone and Periodontal Research Core Facility. The astragalus and calcanuem bone (ankle) volumes were measured. Total porosity and the bone volume to tissue volume ratio (BV/TV) were used to estimate the overall bone loss.

### Stimulation of human neutrophils and quantification of S100A8 homodimers

This study was approved by the CHU de Quebec–Université Laval ethics review board. Written informed content has been obtained from blood donors after the nature and possible consequences of the studies were explained. Patients from the CHU de Québec SARD Biobank Repository Database (SBRD) were included in the study. Plasma from 19 patients suffering from rheumatoid arthritis was obtained between September 2014 and July 2017 at the time of diagnostic (S2 Table). Ten healthy donors were used as controls. Peripheral blood from healthy adult volunteers was collected in heparinized tubes. Neutrophils were isolated as described previously [45] and re-suspended in HBSS supplemented with 10 mM HEPES, pH 7.4 (HBSS-H) containing 1.3 mM Ca^2+^ and 0.8 mM Mg^2+^. Cell purity and viability were consistently > 98% based on acetic blue staining and trypan blue exclusion. Neutrophils (10^7^ cells/mL) were stimulated for 30 min with LPS (100 ng/mL) or its diluent (PBS), then centrifuged and S100A8 homodimers were quantified in the plasma and supernatant by an in-house ELISA described previously [46].

### ELISA

Blood was collected 4 h and 5 days after LPS injection and centrifuged. Serum anti-collagen II antibody titer was measured using an anti-collagen detection kit according to the manufacturer’s instructions (Chondrex inc). Calprotectin, S100A8 and S100A9 were quantified by ELISA as described previously [13]. Murine IL-12, IL-27, IL-6, CXCL1/KC, IL-1β, IL-10 were quantified by multiplex analyses using the mouse cytokine custom premix kit systems according to the manufacturer’s instructions (R&D systems).

### Statistical analyses

S100A8/A9, S100A8, S100A9 concentrations, cartilage, bone destruction, cell infiltration, BV/TV, total porosity, cytokine secretion, cell counts, marker expression, and osteoclastic activity were compared using the unpaired Student t-test in GraphPad Prism version 7.00. Clinical arthritic scores were compared using a Mann-Whitney test. A p value ≤ 0.05 was considered statistically significant.

## Results

### Deletion of S100a8-/- promotes myeloid cell differentiation

**Fig 1:**
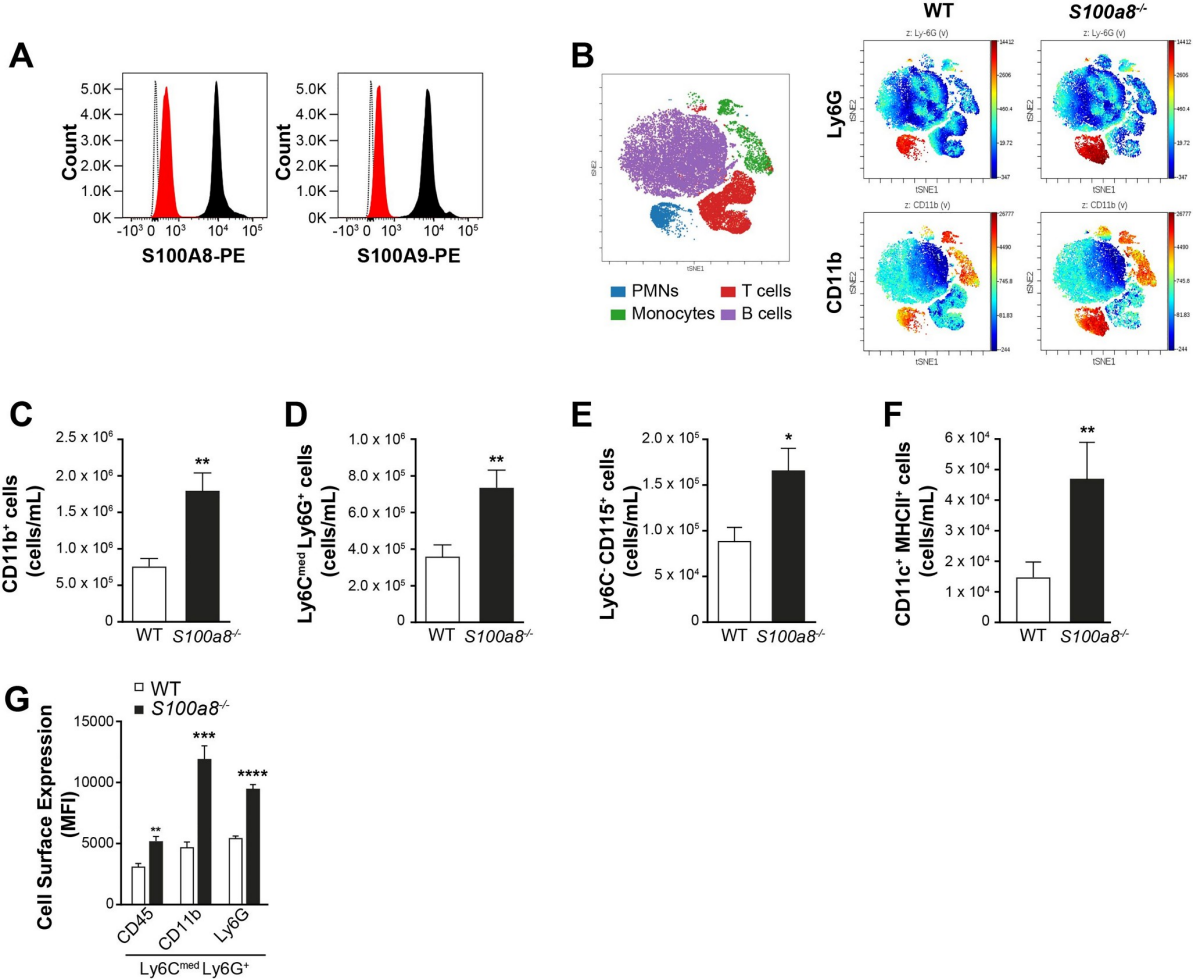
*S100a8*^*-/-*^ mice have increased circulating and mature myeloid cells. (A) Cytometry analysis of S100A8 and S100A9 expression in WT (black) and *S100a8*^*-/-*^ Ly6C^med^Ly6G^+^ (red) blood cells. Dotted line: isotype control antibody in WT cells. Results are from 1 representative experiment (n = 3). (B) Visualization (viSNE) of CD11b and Ly6G expression in leukocyte populations in peripheral blood. Left: representation of cell population distribution, magenta for B cells, green for monocytes, red for T cells and blue for neutrophils. Right: CD11b and Ly6G expression intensity in the different cell populations in WT and *S100a8*^*-/-*^ mice. (C–F) Flow cytometry measurement of: (C) CD11b^+^ myeloid cell counts in peripheral blood, (D) circulating Ly6C^med^Ly6G^hi^ neutrophil counts, (E) circulating Ly6C^hi^Ly6G^low^ monocyte counts, (F) circulating CD11c^+^MHCII^+^ dendritic cells. (G) Surface expression of CD45, CD11b and Ly6G in neutrophils (based on 8 mice). All values are mean ± sem. *p < 0.05; **p < 0.01; ***p < 0.001; ***p < 0.0001, Student’s t-test.

Mice with all *S100a8* coding exons (2 and 3) deleted (S1A Fig) were viable and had normal litter sizes and male-to-female ratios in both the C57BL/6 and the DBA-1 background. Southern blot analyses and DNA sequencing confirmed a single insertion of the DNA cassette (as revealed by the KO probe) and that deletion was restricted to *S100a8*, whereas *S100a9* was not affected (S1B Fig). No S100A8 mRNA was detected in bone marrow cells (S1C Fig) and S100A9 mRNA was present at reduced levels compared to WT. As expected, deletion of *S100a8* led to complete abrogation of S100A8 expression in peripheral blood neutrophils (Fig 1A). Although *S100a9* mRNA was detected in leukocytes, the protein was undetectable (based on ELISA and western blot) and the absence of S100A8 ensured a lack of S100A8/A9 heterodimer in the serum.

Since myeloid cells express S100A8 strongly, we first performed a 13-parameter single-cell flow cytometry analysis combined with visualization using t-distributed stochastic neighbour embedding (viSNE) to study the leukocyte phenotypic diversity that develops in *S100a8*^*-/-*^ mice. Two-dimensional visualization of the multidimensional cytometry data using viSNE revealed an altered geography of the neutrophil cluster in peripheral blood of *S100a8*^*-/-*^ mice. This corresponded to more abundant neutrophils with a higher expression of the myeloid markers CD11b and Ly6G in *S100a8*^*-/-*^ mice (Fig 1B). We thus found altered neutrophil cluster topology in the peripheral blood of *S100a8*^*-/-*^ mice, specifically increased counts of cells with heightened expression of these and other myeloid markers. An increase in the numbers of circulating CD11b^+^ myeloid cells was observed (Fig 1C and S2A Fig), including granulocytes (Ly6C^med^Ly6G^hi^; Fig 1D), non-classical monocytes (Ly6C^-^CD115^+^, Fig 1E), and dendritic cells (CD11c^+^MHC class II^+,^; Fig 1F). Expression of CD45, CD11b and Ly6G was heightened in *S100a8*^*-/-*^ neutrophils (Fig 1G), as expression of CD45 and CD11b was in *S100a8*^*-/-*^ monocytes (S2B Fig). CD4^+^ and CD8^+^ T cells were not affected in the lymphoid organs of these mice (S2C Fig).

**Fig 2:**
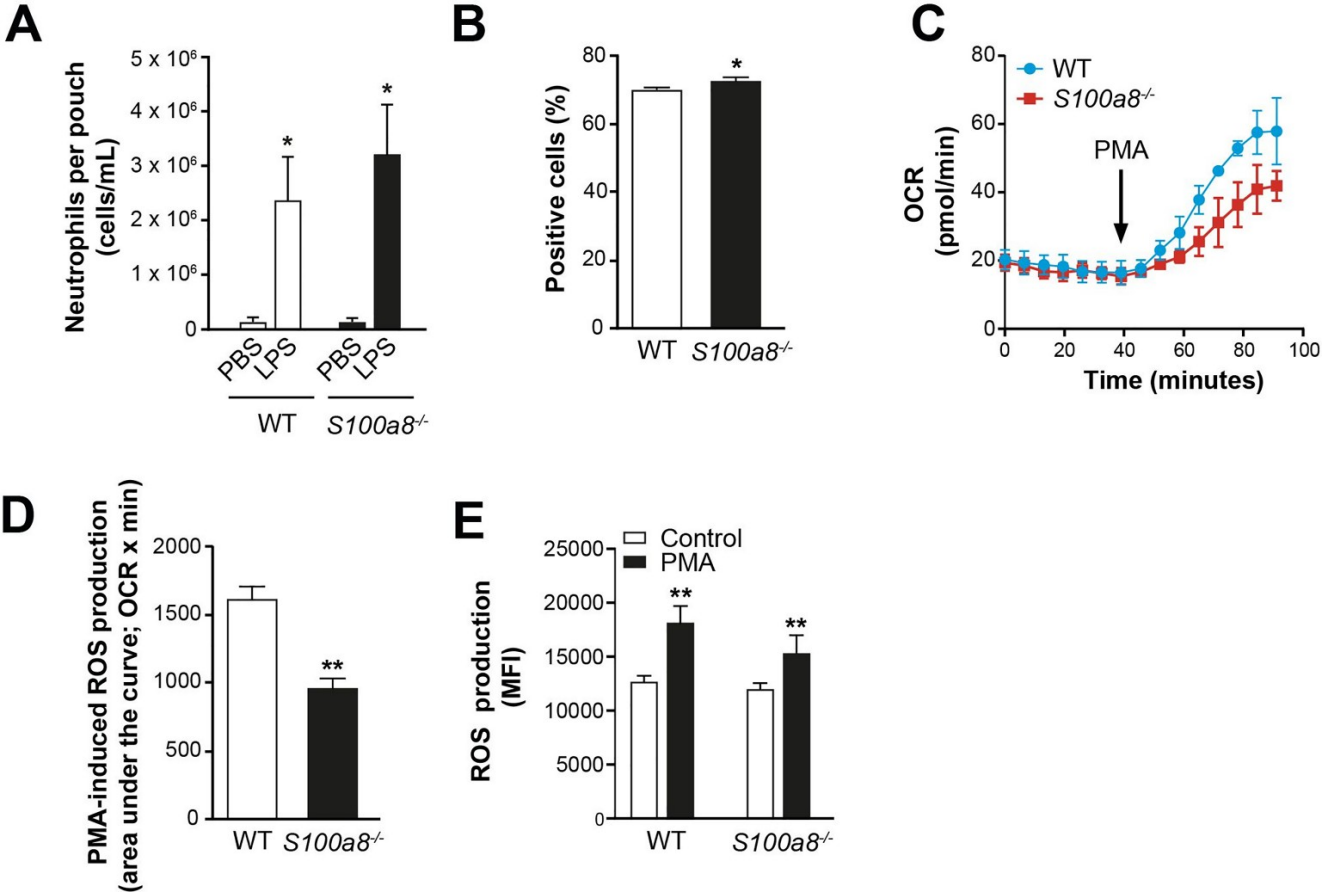
Deletion of *S100a8* gene has limited effects on myeloid cell functions. (A) Neutrophil migration to the dorsal air pouch 6 h after LPS injection (10 mice). (B) Phagocytosis of monocytes. Values are percentage ± sem of fluorescein-labelled monocytes (4 mice). (C, D) Oxygen consumption rate (OCR) of bone marrow neutrophils in response to PMA, based on from 4 sample replicates from 1 representative experiment (out of 3). (E) ROS production measured as oxidation of the fluorogenic probe CellROX by neutrophils in response to PMA as measured by flow cytometry (n = 5). All values are mean ± sem. *p < 0.05; **p < 0.01; ***p < 0.001; ***p < 0.0001, Student’s t-test.

The integrin CD11b is involved in myeloid cell migration and acts as a major opsonin receptor. To investigate the effect of *S100a8* gene deletion on neutrophil and monocyte migration, bacterial lipopolysaccharide (LPS) was injected into air pouches raised on the dorsum of WT and *S100a8*^*-/-*^ mice. This induced unequivocal neutrophil migration to the air pouch, although the responses of the two mouse strains did not differ significantly (Fig 2A). Intra-peritoneal injection of thioglycolate also induced similar inflammatory responses in both strains (S2D Fig). This suggests that CD11b up-regulation in *S100a8*^*-/-*^ mice does not affect intrinsic migration of myeloid cells. We next examined the effect of heightened expression of CD11b on phagocytosis in *S100a8*^*-/-*^ myeloid cells. *S100a8*^*-/-*^ monocytes were slightly more inclined than WT monocytes to engulf bacteria (Fig 2B), but cells from both genetic backgrounds engulfed similar numbers. The heightened expression of CD11b thus affords a slight improvement of myeloid cell phagocytic activity.

A hallmark of neutrophils and monocytes is ROS production, and it has been suggested that S100A8/A9 regulates ROS accumulation. Since S100A8 is easily oxidized, it could scavenge ROS [47]. In addition, S100A8/A9 activates phagocyte NADPH oxidase by transferring to it the co-factor arachidonic acid [48]. We therefore analyzed oxygen consumption by *S100a8*^*-/-*^ neutrophils using an extracellular flux analyzer. Neutrophils have very few mitochondria and almost no mitochondrial respiration. Most of their oxygen consumption is associated with NADPH oxidase activity, as demonstrated by inhibition of PMA-induced oxygen consumption using DPI (S3 Fig). Therefore, analysis of oxygen consumption by neutrophils allowed us to directly interrogate NADPH oxidase activation, without possible interference from ROS scavenging by S100A8. Direct measurement of NADPH oxidase activity revealed that oxygen consumption in response to PMA stimulation was delayed in *S100a8*^*-/-*^ neutrophils (Fig 2C), with 40% less oxygen consumed over a period of 50 min (Fig 2D). Flow cytometry revealed a corresponding reduction of 39% in ROS produced by *S100a8*^*-/-*^ neutrophils (Fig 2E). These results suggest that absence of S100A8 leads to inefficient NADPH oxidase activity in neutrophils, and that scavenging of ROS by S100A8 is limited.

### Deletion of S100a8 promotes production of dendritic cells by bone marrow

**Fig 3:**
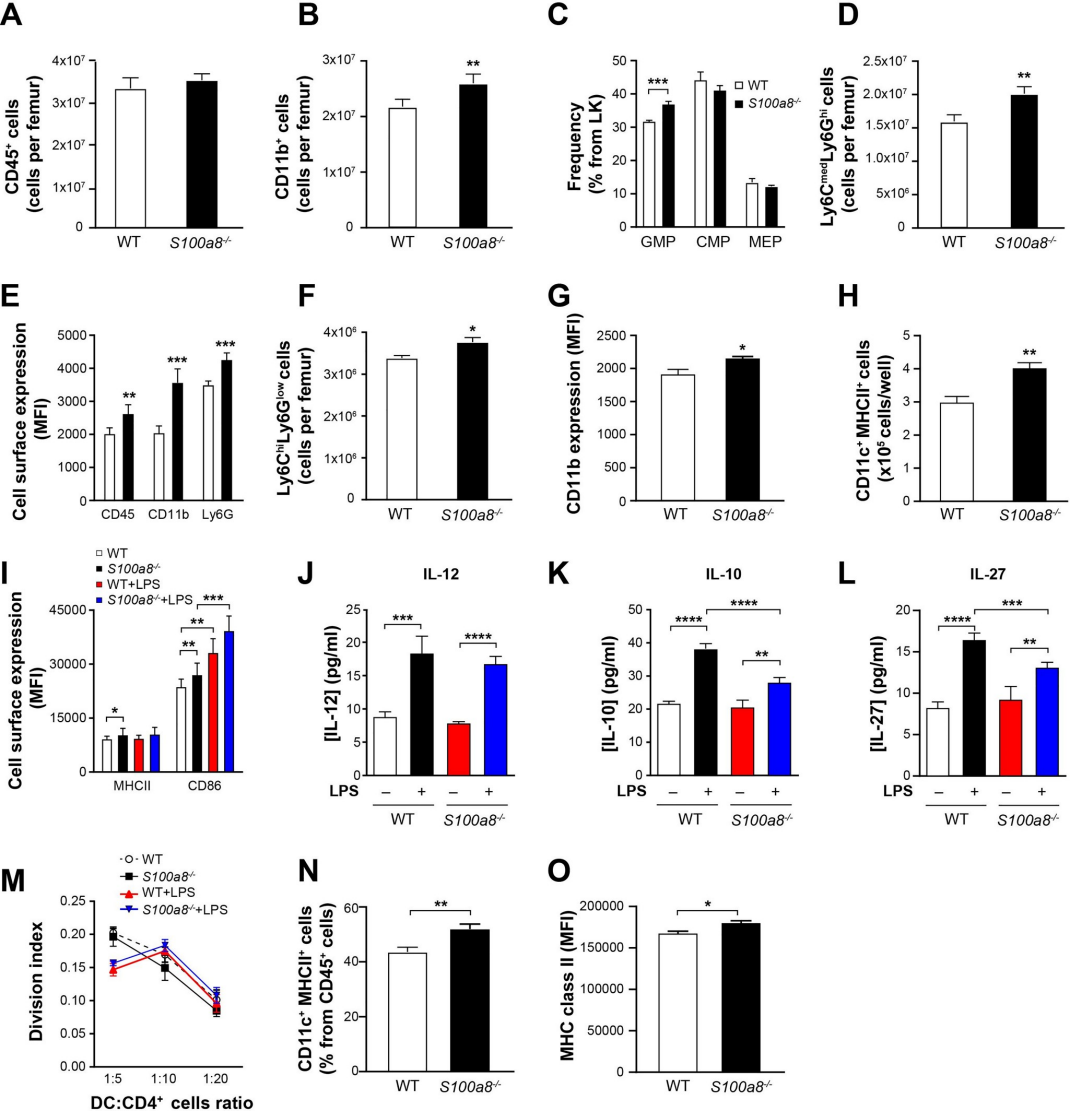
Increased numbers of dendritic cells in *S100a8*^*-/-*^ mice. (Values in A–I are from flow cytometry, based on 4 mice in A–G and on 12 mice in H and I. WT = wild type.) (A) CD45+ cells in bone marrow. (B) CD11b^+^ myeloid cells in bone marrow. (C) Frequency of granulocyte-macrophage progenitors (GMP), common monocyte progenitors (CMP) and megakaryocyte-erythrocyte progenitors (MEP) in bone marrow. (D) Neutrophil-committed (Ly6C^med^Ly6G^hi^) cells in bone marrow. (E) Expression of CD45, CD11b and Ly6G on the surface of neutrophil-committed cells. (F) Monocyte-committed (Ly6C^hi^Ly6G^low^) cells in bone marrow (G) Expression of CD11b on the surface of monocyte-committed cells. (H) Bone-marrow-derived dendritic cells (CD11c^+^MHC class II^+^) cultured with GM-CSF and IL-4. (I) Expression of MHC class II (MHCII) and CD86 on the surface of dendritic cells. (J–L) Secretion of IL-12, IL-10 and IL-27 by dendritic cells stimulated with 100 ng/mL LPS (4 mice). (M) Dendritic-cell-induced proliferation (mixed lymphocyte reaction) of CD4^+^ T cells obtained from B10 mice versus DC/CD4^+^ T ratio (3 mice). (N) Dendritic cell crawl-out from the skin (8 mice). (O) Expression of MHC class II molecules on the surface of dendritic cells crawling out of ear skin (8 mice). All values are mean ± sem. *p < 0.05; **p < 0.01; ***p < 0.001, Student’s t-test.

The larger number of circulating neutrophils, monocytes and dendritic cells in *S100a8*^*-/-*^ mice suggested that the deletion alters myeloid cell production by bone marrow. While total numbers of leukocytes were similar in bone marrows of WT and *S100a8*^*-/-*^ mice (Fig 3A), analyses of bone marrow cells revealed that CD11b^+^ myeloid and granulocyte monocyte progenitor (GMP) populations were increased respectively by 22% and 19% compared to WT mice (Fig 3B and 3C; S4A Fig). In contrast, no differences in the numbers of megakaryocyte-erythroid progenitor (MEP), common monocyte progenitor (CMP), monocyte-macrophage DC progenitor (MDP) and common monocyte progenitor (cMOP) numbers were detected (Fig 3C; S4A–S4C Fig). Bone marrow of *S100a8*^*-/-*^ mice contained 33% more neutrophil-committed cells (Ly6C^med^Ly6G^hi^, Fig 3D), and these expressed higher levels of CD45, CD11b and Ly6G compared to those from WT mice (Fig 3E). Similarly, monocyte-committed cells were slightly elevated in *S100a8*^*-/-*^ bone marrow (Fig 3F) and expressed more CD11b than did those from WT (Fig 3G). These results suggest that *S100a8* acts as a repressor of myeloid cell differentiation in bone marrow.

We next derived dendritic cells from bone marrow to determine if the absence of S100A8 leads to greater numbers of DC precursors. Using GM-CSF and IL-4, approximately 35% more dendritic cells were generated in the case of *S100a8*^*-/-*^ (Fig 3H), and these expressed more MHC class II and CD86 (Fig 3I), suggesting a more mature phenotype. Stimulation with LPS increased expression of CD86 but not MHC class II in dendritic cells derived from either mouse strain. Secretion of IL-12 following activation by LPS was also similar, whereas concentrations of immunosuppressive cytokines IL-10 and IL-27 were reduced for *S100a8*^*-/-*^ DCs (Fig 3J–3L). However, *S100a8*^*-/-*^ DCs were as efficient as WT DCs in terms of inducing CD4^+^ lymphocyte proliferation (Fig 3M). More dendritic cells were also found in *S100a8*^*-/-*^ skin, as revealed by cell crawl-out observations (Fig 3N), and these cells also exhibited increased expression of MHC class II (Fig 3O). The absence of *S100a8* thus leads to increased differentiation and activation of myeloid cells in granulocytes, monocytes and dendritic cells.

### Aggravated arthritis in S100a8-/- mice

**Fig 4:**
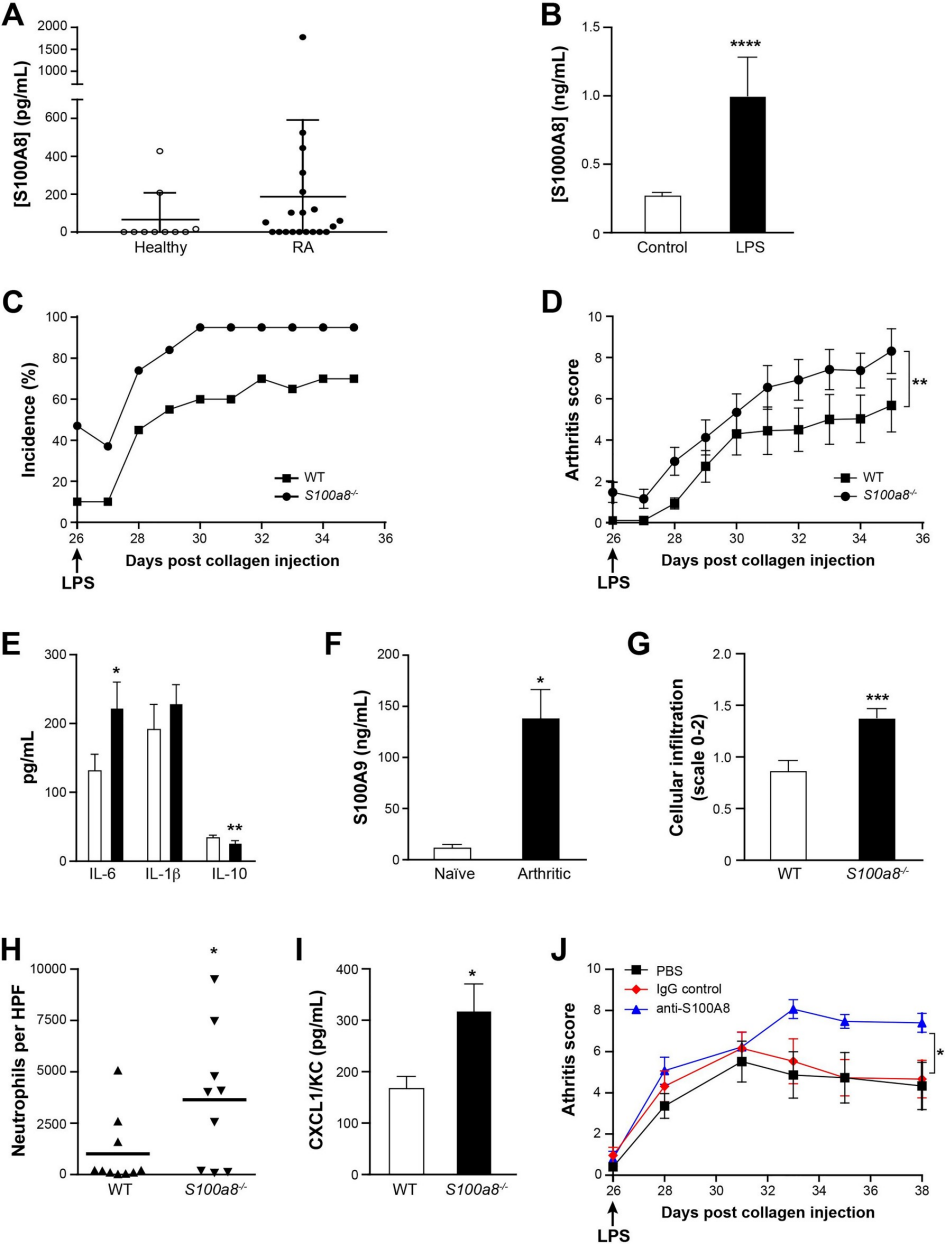
S100A8 mitigates arthritis. (A) Presence of S100A8 homodimer in the plasma of 20 rheumatoid arthritis patients and 10 age-matched and sex-matched healthy donors. S100A8 was quantified using an in-house ELISA. (B) Secretion of S100A8 homodimer by human peripheral blood neutrophils stimulated with LPS (100 ng/mL). S100A8 in culture supernatant was quantified using an in-house ELISA (12 healthy donors). (C) Incidence of collagen-induced arthritis in WT and *S100a8*^*-/-*^ mice. (D) Arthritis clinical score as measured by impartial observers (n = 20 for WT and n = 19 for *S100a8*^*-/-*^; ** based on GEE analysis). (E) Expression of IL-6, IL-1β and IL-10 in paws of WT and *S100a8*^*-/-*^ mice (n = 18). (F) Expression of S100A9 in the paws of naïve and arthritic *S100a8*^*-/-*^ mice (n = 4 naïve and n = 19 arthritic). (G) Histological assessment of cellular infiltration into joints (scale of 0–2) as assessed by two impartial observers (n = 20 for WT and n = 19 for *S100a8*^*-/-*^). (H) Neutrophil infiltration into joints of arthritic WT and *S100a8*^*-/-*^ mice. Neutrophils were enumerated in histological slides of arthritic paws (n = 9). (I) CXCL1/KC expression in homogenates of paws as assessed by multiplex assays (n = 18 paws). (J) Effect of anti-S100A8 IgG on collagen-induced arthritis Anti-S100A8 IgGs or normal rabbit IgG was injected 3 times per week starting on the day of LPS injection (n = 20 for WT and n = 19 for *S100a8*^*-/-*^; * based on GEE analysis). All values are mean ± sem. *p < 0.05; **p < 0.01; ***p < 0.001, Student’s t-test.

Elevated concentrations of calprotectin are found in the plasma and synovial fluids of rheumatoid arthritis patients, and S100A9 is known to exacerbate chronic inflammation in models of arthritis [6, 24, 49, 50]. In contrast, increased numbers of myeloid cells including neutrophils, monocytes and dendritic cells suggest that immune responses might be aggravated in the absence of the *S100a8* gene. However, data on the distribution of the S100A8 homodimer are scarce, and before investigating its possible role in arthritis, we first sought to determine if it is even present in association with the disease. We found it in plasma obtained from healthy donors and patients with rheumatoid arthritis (Fig 4A). We then confirmed in vitro that human neutrophils secrete small amounts of S100A8 homodimers spontaneously, and that this secretion is accentuated following stimulation with LPS (Fig 4B). To confirm that the adaptive immune response is enhanced in *S100a8*^*-/-*^ mice, arthritis was induced by injection of chicken collagen type II once at the base of the tail. This was sufficient to induce a rapid and strong response within 25 to 30 days, which was then inflamed with LPS. This led to arthritis in respectively 60% and 95% of WT and *S100a8*^*-/-*^ mice (Fig 4C), the latter developing more severe disease, with a mean score of 8.3 ± 1.1 on day 35, compared to 5.7 ± 1.3 for WT (Fig 4D). While expression of IL-6 was increased by 68% and IL-1β expression was similar, expression of anti-inflammatory cytokine IL-10 was 14% lower in the paw homogenate of *S100a8*^*-/-*^ mice (Fig 4E), suggesting that the immune response in the absence of S100A8 is skewed toward a pro-inflammatory outcome. S100A9 was detected at 13.05 ± 3.85 ng/mL in the paws of naïve *S100a8*^*-/-*^ mice and about 10 times higher in arthritic paws (Fig 4F), which was nowhere near the level measured in WT animals (about 4,000 ng/mL). Deletion of the *S100a8* gene thus markedly reduced the S100A9 concentration reached in arthritic mice, although it did not eliminate it. Analyses of hematoxylin/eosin stains of paw tissue sections showed more extensive cell infiltration into the paws of *S100a8*^*-/-*^ mice (Fig 4G), with significantly more Ly6G^+^ neutrophils in the metatarsal joints (Fig 4H). This was associated with increased concentrations of the neutrophil chemo-attractant CXCL1/KC (Fig 4I). These results indicate that absence of S100A8 aggravates inflammation in arthritic *S100a8*^*-/-*^ mice.

S100A8 exerts intracellular and extracellular activities. To provide additional support for the idea that the increased disease severity observed in S100A8-deficient mice is due to the absence of extracellular S100A8, arthritis was induced in WT mice treated with anti-S100A8, a control antibody or PBS. Injection of anti-S100A8 IgG increased the arthritis score (Fig 4J), indicating that extracellular S100A8 provides some protection against chronic inflammation.

### Increased cartilage and bone destruction in S100a8-/- arthritic mice

**Fig 5:**
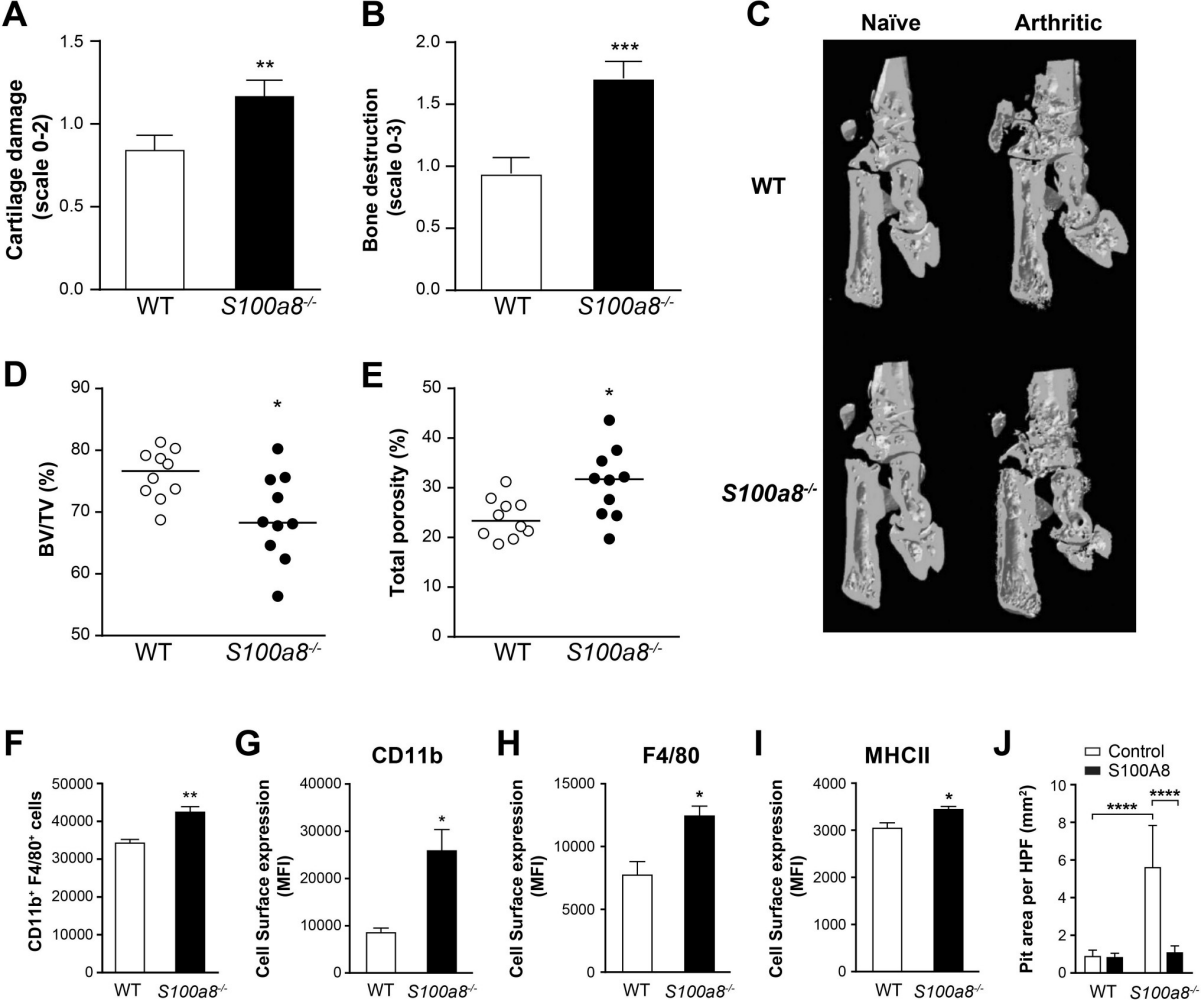
Increased bone destruction and osteoclast activity in *S100a8*^*-/-*^ mice with collagen-induced arthritis. (A) Cartilage degradation in paws, histological scale of 0–2 and (B) bone destruction, histological scale of 0–3, as assessed by two impartial observers (n = 20 for WT and n = 19 for *S100a8*^*-/-*^ mice). (C) 3D surface renderings showing bone erosion as assessed by μCT scans of paws. One hind paw representative of 9 is shown. (D) Bone volume/tissue volume and (E) total porosity of the astragalus bone of WT and *S100a8*^*-/-*^ arthritic mice. Dots are individual values (bar = mean of 10 measurements). (F) Cultured *S100a8*^-/-^ bone marrow cells stimulated with GM-CSF produce more macrophages. The cells were harvested, counted and analyzed by flow cytometry after 6 days. (G–I) Expression of CD11b, F4/80 and MHC class II is increased on the surface of bone marrow-derived macrophages from *S100a8*^-/-^ mice compared to WT mice, based on flow cytometry (3 mice, one representative experiment out of 3). (J) Osteoclastic activity of *S100a8*^-/-^ bone-marrow-derived cells (obtained by incubation with M-CSF and RANKL for 10 days in osteoassay plates) in the presence of extracellular S100A8. Pit areas were measured by microscopy. Two experiments were performed on cells from different mice. All values are mean ± sem. *p < 0.05; **p < 0.01; ***p < 0.001, Student’s t-test.

Increased damage to cartilage and bone structure was observed in arthritic *S100a8*^*-/-*^ animals (Fig 5A and 5B). Histomorphometric analysis of the astragalus bone by μCT revealed more asperities and a marked loss of mass compared to WT mice. Typical examples are shown in Fig 5C. The average bone volume to tissue volume ratio (BV/TV) was lower in *S100a8*^*-/-*^ mice (69.1 ± 2.2% versus 76.1 ± 1.3% in WT mice, Fig 5D) and the average total porosity was higher (30.8% ± 2.2% versus 23.9% ± 1.3%, Fig 5E). These results indicate that the surface of mineralized bone is reduced in *S100a8*^*-/-*^ compared to WT mice and that the bones are more degraded.

Osteoclasts derived from monocytes are presumed to cause bone resorption in rheumatoid arthritis [51]. To determine if S100A8 inhibits the differentiation of osteoclasts, bone marrow cells were cultured in the presence of GM-CSF to generate bone-marrow-derived macrophages. This yielded 25% more macrophages in the *S100a8*^*-/-*^ case (Fig 5F). As observed in circulating monocytes, bone-marrow-derived macrophages from these mice expressed higher levels of CD11b, F4/80 and MHC class II (Fig 5G–5I). Bone marrow cells were then cultured in presence of M-CSF and RANKL in wells coated with a mineralized matrix to generate osteoclasts. The area resorbed by these osteoclasts was three times larger compared to that of bone marrow cells from WT animals (Fig 5J). This observation corroborates the increased porosity observed in *S100a8*^*-/-*^ animals and indicates that their osteoclast activity is increased. Adding S100A8 nullified the increase in resorption, indicating that extracellular S100A8 regulates osteoclast numbers and activities. These results suggest that the gene deletion in *S100a8*^*-/-*^ mice leads to an increase in osteoclast activation and ultimately to significant bone degradation once arthritis is induced.

## Discussion

Despite decades of research, the biological activity of S100A8 remains poorly understood. Upon discovery, this protein was first found to be a potent chemotactic factor for neutrophils and monocytes [9, 25]. Subsequent study indicated that upon oxidation, it acts as an anti-inflammatory factor inhibiting mast cell activation [33, 34, 52]. Recent reports suggest that it promotes the commitment of hematopoietic stem cells to the myeloid lineage and inhibits the differentiation of acute myeloid leukemia cells [18, 35]. In this study, we report the characterization of the first viable *S100a8*^*-/-*^ mouse strain. Deletion of the *S100a8* gene increases the numbers of granulocyte and monocyte progenitors and promotes the differentiation of mature myeloid cells including neutrophils, monocytes and dendritic cells. It has no effect on acute inflammation but does aggravate induced arthritis. Exacerbated disease is associated with the marked presence of mononuclear phagocytes, pro-inflammatory factors including S100A9 and cytokines, and osteoclastic activity potentiating joint damage and bone degradation in the paws. These results suggest that S100A8 has a damping effect on chronic inflammation by regulating myeloid cell differentiation.

Disruption of the *S100A8* gene in exon 2 in mice is reportedly lethal during early development [39, 40]. In one study, homozygous null embryos (*S100a8*^*-/-*^) were resorbed by day 9.5 in utero [39, 40], while another study reported failure of zygotes to develop beyond day 2.5 [39]. In sharp contrast, we report a viable *S100a8*^*-/-*^ strain generated by deleting all *S100a8* coding exons. No embryo resorption has been observed to date. The absence of S100A8 in the KO mice has been confirmed at the genomic, RNA and protein levels using Southern blot, RT-PCR and western blot analyses. The reason for the viability of these KO mice is not clear. The previous studies mention deletion of most or all of exon 2, which is the first coding exon of the *S100a8* gene. Since exon 3 was not targeted for deletion, it is possible that a spliced version of *S100a8* was formed around the selection cassette in these ES cells. This could lead to translation of a protein truncated to 16 amino acids using the strong in-frame ATG start codon in exon 3 that could have biological activities. By deleting the entire coding portion of the *S100a8* gene, no truncated version of this protein can be formed. Like *S100a9*^*-/-*^ mice, in which S100A8 is weakly expressed [53] or not detectable [54] in bone marrow cells, *S100a8*^*-/-*^ mice do not express S100A8 or S100A9 proteins in bone marrow and peripheral blood leukocytes in the absence of inflammation. However, S100A9 was detected in *S100a8*^*-/-*^ mice under inflammatory conditions, indicating that S100A8 and S100A9 can be expressed independently.

The generation of viable *S100a8*^*-/-*^ mice offers new perspectives for the study of S100A8 and S100A9, and the comparison of responses developed in *S100a8*^*-/-*^ and *S100a9*^*-/-*^ mice during inflammatory processes could help define the functions of the S100A8/A9 heterodimer. We cannot rule out the possibility that the phenotypic effects observed in *S100a8*^*-/-*^ mice are due to the loss of S100A8/A9 or S100A9. However, most of the activities reported for the heterodimer are also attributed to the S100A9 homodimer and accumulating evidence strongly indicates that S100A9 is a major pro-inflammatory factor [12–14, 22, 36]. Experimental arthritis is less severe in *S100a9*^*-/-*^ mice (which lack S100A9 and S100A8/A9 proteins) [6] and in mice receiving anti-S100A9 antibodies [13]. In contrast, it is aggravated in mice lacking S100A8 and S100A8/A9 (this study). These studies suggest that S100A9 is pro-inflammatory whereas S100A8 is anti-inflammatory in experimental arthritis. In addition, since S100A8/A9 is absent in both genotypes, these data suggest that the role of S100A8/A9 is not as important as that of the homodimers.

The hallmark of the *S100a8*^*-/-*^ mouse is the increase in myeloid cell counts in peripheral blood and bone marrow. Massive cellular multiplication occurs during the lineage maturation that leads to the generation of neutrophils, monocytes and dendritic cells [55]. However, with the notable exception of GMP, progenitors and precursors of myeloid cells are not increased in bone marrow of *S100a8*^*-/-*^ mice, suggesting that S100A8 regulates the differentiation, but not the proliferation of myeloid cells. This is in contrast with a recent report indicating that S100A8 promotes changes in hematopoietic stem cells, supporting myeloid skewing in experimental arthritis [35] and possibly a positive feedback loop in which secreted S100A8 triggers emergency myelopoiesis, which cannot occur in a true *S100a8*^*-/-*^ mouse. We demonstrated recently that S100A8 regulates differentiation of myeloid cells associated with acute leukemia, a disease characterized by overabundance of myeloid cell precursors and progenitors [18]. These studies show that S100A8 and S100A9, both produced by acute myeloid leukemia cells, regulate the differentiation and proliferation of myeloid cell precursors and progenitors. Modifying the S100A8/S100A9 ratio by blocking S100A8 with antibodies or adding S100A9 induces the differentiation of acute myeloid leukemia cells and their growth arrest in mouse and human models. Again, these studies highlight the opposite effects of S100A8 and S100A9, the former inhibiting differentiation and the latter promoting the maturation and differentiation of precursors and progenitors of myeloid cells.

Dendritic cells were detected in greater numbers in the blood and skin of *S100a8*^*-/-*^ mice, indicating increased production. Myeloid cell maturation is linked with the silencing of *S100a8* and *S100a9* expression, and neither mRNA is expressed in macrophages or mature dendritic cells, while overexpression of both proteins leads to inhibition of macrophage and dendritic cell differentiation [56]. It is interesting that dendritic cells from *S100a9*^*-/-*^ mice exhibit an increased pro-inflammatory response and are stronger inducers of T cell proliferation, suggesting that they are more mature [57]. Through more efficient antigen presentation, the increased abundance of dendritic cells alone could be a major contributor to the aggravated disease observed in *S100a8*^*-/-*^ mice.

The onset of rheumatoid arthritis involves an aberrant activation of auto-reactive B and T cells by dendritic cells, giving rise to Th1/Th17 responses and increased activity of neutrophils, macrophages and osteoclasts, leading to bone and cartilage degradation [58]. S100A8 was first described as a 10 kDa chemotactic protein (CP-10), a potent chemotactic factor that attracts neutrophils at concentrations in the 10^−11^ to 10^−13^ M range [22, 59]. Neutrophils stimulated with LPS release S100A8 at low concentrations (10^−10^ M), and the concentrations found in the plasma of patients with arthritis (10^−11^ M) are consistent with its chemotactic activity [60]. Antibodies against S100A8 also reduce cell migration to the dorsal air pouch in response to inflammation induced by LPS [23] or by monosodium urate crystals [22] and to the lungs in response to *Streptococcus pneumoniae* infection [12]. It was therefore unexpected that deletion of *S100a8* had no effect on myeloid cell migration in two models of acute inflammation. This suggests a compensatory mechanism in *S100a8*^*-/-*^ mice, which could be due to the increased numbers of circulating myeloid cells. In contrast, the observed increase in neutrophil and macrophage infiltration in arthritis likely resulted from indirect enhancement of the inflammatory response through altered cytokine secretion. S100A8 appears to induce expression of the anti-inflammatory cytokine IL-10 [28], which was found down-regulated in arthritic *S100a8*^*-/-*^ mice. In addition, the pro-inflammatory chemokine CXCL1/KC and cytokine IL-6 were elevated in these mice. Oxidized S100A8 is a known inhibitor of mast cell degranulation and FcεR-crosslinking-induced cytokine secretion [31, 33, 47, 61], and at least two studies indicate that it has an anti-inflammatory role in acute asthma and sepsis [33, 52], which is attributed to inhibition of inflammatory pathways mediated by ROS. Together, these observations suggest strongly that S100A8 acts as an anti-inflammatory factor in chronic inflammation.

Another characteristic of *S100a8*^*-/-*^ mice is the enhanced osteoclastic activity in the arthritic paws. This observation was corroborated with μCT analyses revealing more porous and degraded bones. S100A8 has been reported to participate in osteoclast formation and activation in *S100a9*^*-/-*^ mice [38], which express S100A8 in some bone marrow cells [53] and at the inflammatory site (our unpublished observations). Since extracellular S100A8 nullifies the effects of the gene deletion on osteoclast production, it likely affects osteoclast differentiation and activity directly by binding to an unknown receptor.

## Conclusions

The results of this study suggest that S100A8 is a major regulator of myelopoiesis and that it plays an anti-inflammatory role. More importantly, this study reiterates that the simple release of intracellular proteins into the inflammatory milieu does not guarantee that they act as alarmins. In fact, S100A8 has been shown to inhibit mast cell activation, scavenge ROS and decrease arthritis symptoms and thus may be considered a potent anti-inflammatory factor. In view of these results, we suggest that it would be helpful to subdivide DAMPs into at least two categories, namely “alarmins” and “dampenins”, S100A8 being a prime example of the latter.

## Supporting information

S1 Fig Targetted disruption of *S100a8* gene. (A) Targeting strategy used to disrupt the *S100a8* locus. Homologous recombination between the target vector and the *S100a8* gene results in the replacement of exons 2 and 3 with the selection cassette. (B) Gel electrophoretic resolution of PCR products generated from amplified mouse-tail DNA, revealed by ethidium bromide fluorescence on 1% agarose. (C) Gel electrophoretic resolution of RT-PCR products generated from bone marrow RNA (ethidium bromide fluorescence, on 2% agarose).
(TIF)

S2 Fig Analysis of leukocyte subsets in *S100a8*^*-/-*^ mice. (A) Gating strategy used in flow cytometry to analyse different subsets of circulating leukocytes: 1, 2 and 3 are respectively neutrophils (CD45^+^CD11b^+^Ly6C^med^Ly6G^+^), Ly6C^+^ monocytes (CD45^+^CD11b^+^Ly6C^high^Ly6G^-^ cells) and dendritic cells (CD45^+^CD11b^+^Ly6G^-^Ly6C^-^CD11c^+^MHCII^+^). (B) Cell surface expression of CD45 and CD11b on peripheral blood monocytes (Ly6C^hi^Ly6G^low^) from WT and *S100a8*^*-/-*^ mice (n = 8). (C) Percentage of CD4^+^ and CD8^+^ cells among CD3^+^TCRα/β^+^ cells in the thymus, spleen and lymph nodes of WT and *S100a8*^*-/-*^ mice, based on flow cytometry (n = 10). (D) Leukocyte migration to the peritoneum in response to thioglycolate in WT and *S100a8*^*-/-*^ mice. Leukocytes were recovered 4 h after intra-peritoneal injection of thioglycolate or PBS (n = 3 or 8).
(TIF)

S3 Fig Oxygen consumption in murine neutrophils is almost completely dependent on NADPH oxidase. Oxygen consumption rate in response to PMA stimulation of neutrophils purified by negative selection from bone marrows of WT and *S100a8*^*-/-*^ mice was quantified using an extracellular flux analyzer. Neutrophils were incubated in the presence or absence of 5 μM of the NADPH oxidase inhibitor DPI. Values are mean ± sem for 4 wells from one experiment representative of 3.
(TIF)

S4 Fig Analysis of bone marrow cells in *S100a8*^*-/-*^ mice. (A) Gating strategy used in flow cytometry to detect GMP (Lin^-^Sca1^-^cKit^+^CD16/32^high-med^CD34^+^), CMP (Lin^-^Sca1^-^cKit^+^CD16/32^med-low^CD34^+^ cells) and MEP (Lin^-^Sca1^-^cKit^+^CD16/32^low^CD34^-^) cells. (B) Flow cytometry gating strategy used to detect MDP (R1, CD117^+^CD115^+^CD135^+^Ly6C^-^CD11b^-^) and cMop (R2, CD117^+^CD115^+^CD135^-^Ly6C^+^CD11b^-^) cells. C) Percentages of MDP and cMop cells in WT and *S100a8*^*-/-*^ bone marrows (n = 6).
(TIF)

S1 Table List of antibodies used in this study. (DOCX)

S2 Table Demographic and clinical data of the research project participants. (DOCX)
