# Supplementary figures and images for: Enhanced myelopoiesis and aggravated arthritis in S100a8-deficient mice
Source: PLoS One. 2019 Aug 22;14(8):e0221528. doi: 10.1371/journal.pone.0221528 (PMC6705798; doi:10.1371/journal.pone.0221528)

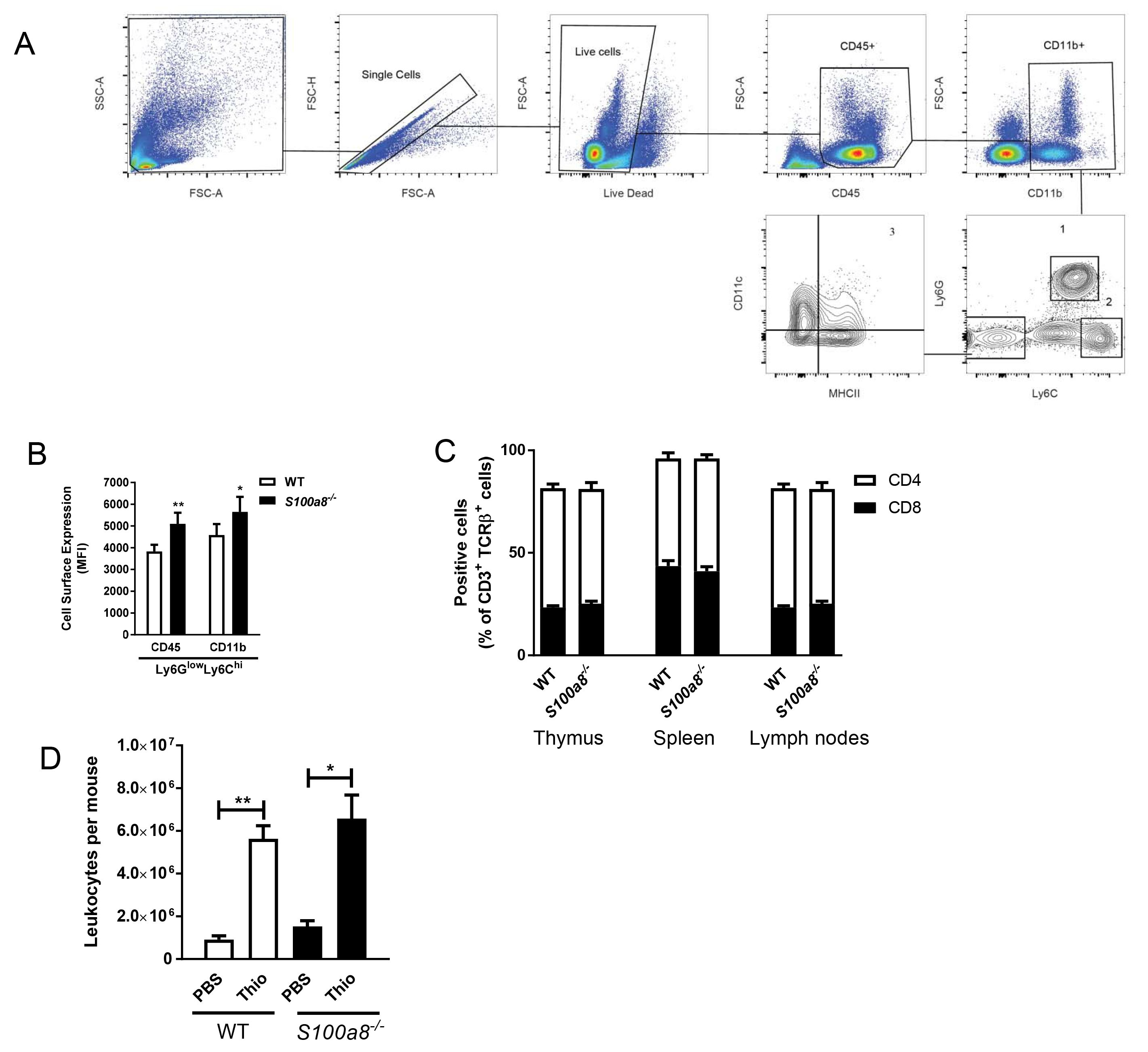

Supplement: S2 Fig — (A) Gating strategy used in flow cytometry to analyse different subsets of circulating leukocytes: 1, 2 and 3 are respectively neutrophils (CD45+CD11b+Ly6CmedLy6G+), Ly6C+ monocytes (CD45+CD11b+Ly6ChighLy6G- cells) and dendritic cells (CD45+CD11b+Ly6G-Ly6C-CD11c+MHCII+). (B) Cell surface expression of CD45 and CD11b on peripheral blood monocytes (Ly6ChiLy6Glow) from WT and S100a8-/- mice (n = 8). (C) Percentage of CD4+ and CD8+ cells among CD3+TCRα/β+ cells in the thymus, spleen and lymph nodes of WT and S100a8-/- mice, based on flow cytometry (n = 10). (D) Leukocyte migration to the peritoneum in response to thioglycolate in WT and S100a8-/- mice. Leukocytes were recovered 4 h after intra-peritoneal injection of thioglycolate or PBS (n = 3 or 8). (TIF) [file pone.0221528.s002.tif]

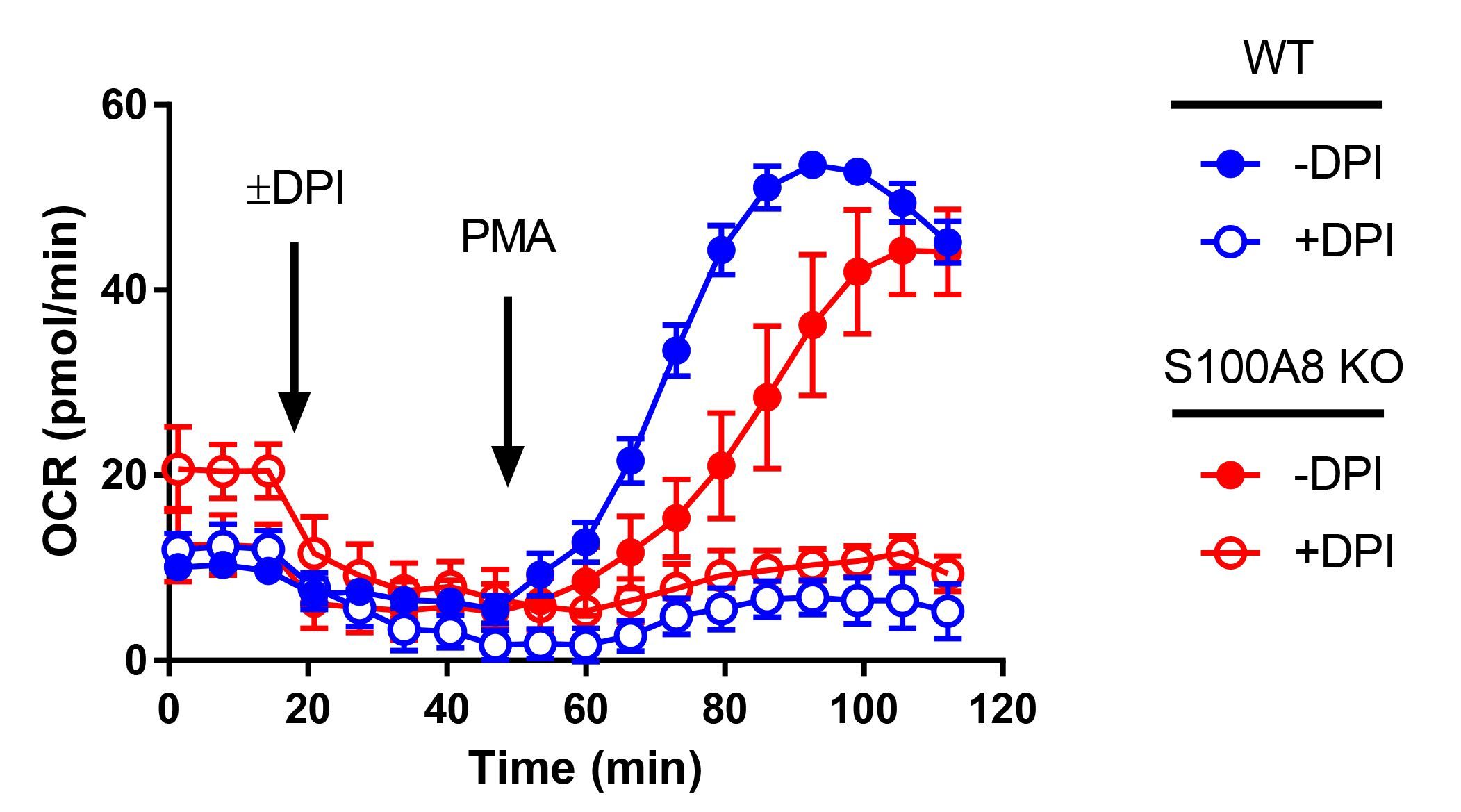

Supplement: S3 Fig — Oxygen consumption rate in response to PMA stimulation of neutrophils purified by negative selection from bone marrows of WT and S100a8-/- mice was quantified using an extracellular flux analyzer. Neutrophils were incubated in the presence or absence of 5 μM of the NADPH oxidase inhibitor DPI. Values are mean ± sem for 4 wells from one experiment representative of 3. (TIF) [file pone.0221528.s003.tif]

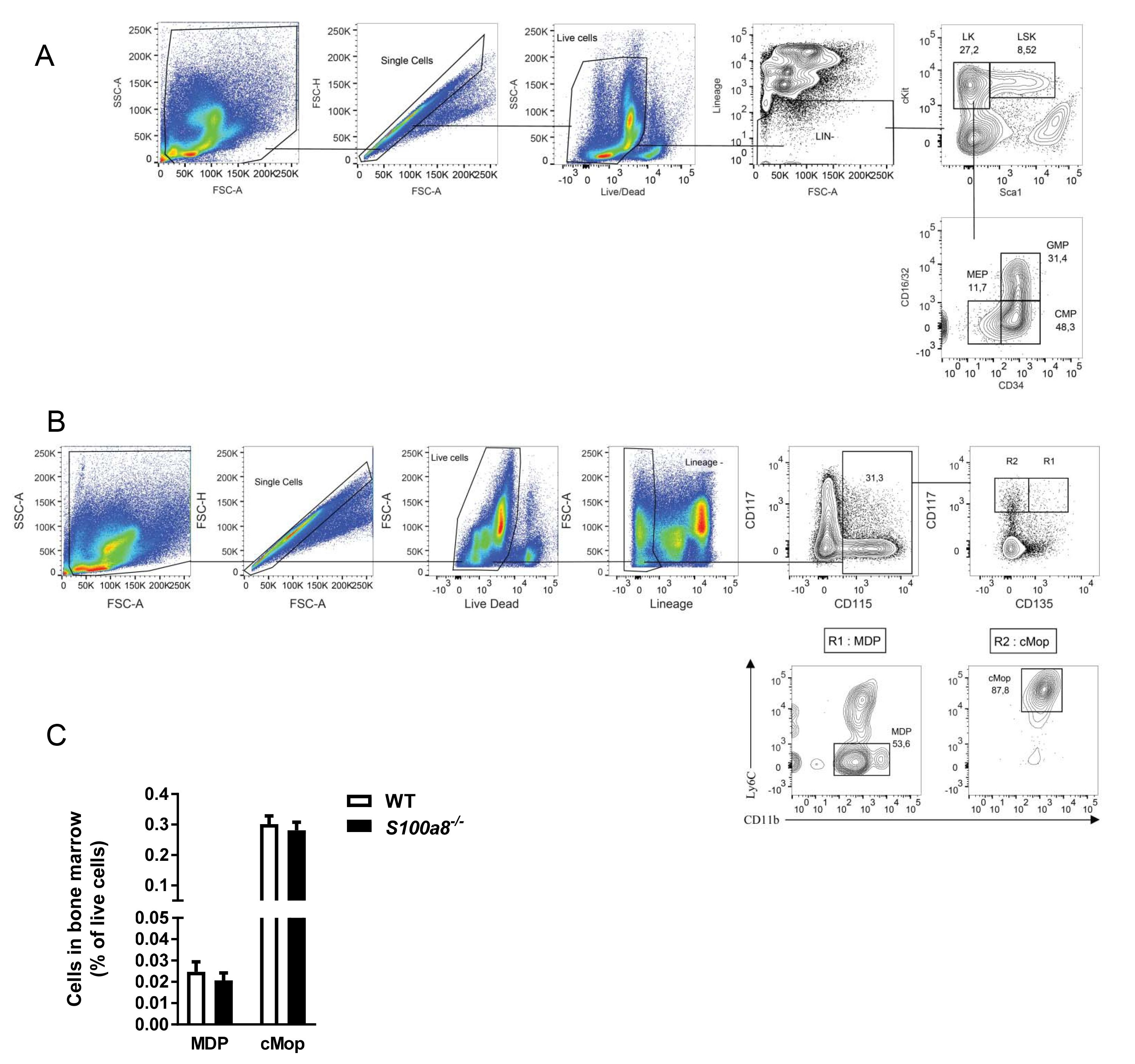

Supplement: S4 Fig — (A) Gating strategy used in flow cytometry to detect GMP (Lin-Sca1-cKit+CD16/32high-medCD34+), CMP (Lin-Sca1-cKit+CD16/32med-lowCD34+ cells) and MEP (Lin-Sca1-cKit+CD16/32lowCD34-) cells. (B) Flow cytometry gating strategy used to detect MDP (R1, CD117+CD115+CD135+Ly6C-CD11b-) and cMop (R2, CD117+CD115+CD135-Ly6C+CD11b-) cells. C) Percentages of MDP and cMop cells in WT and S100a8-/- bone marrows (n = 6). (TIF) [file pone.0221528.s004.tif]
