# Supplementary material for: Enhanced myelopoiesis and aggravated arthritis in S100a8-deficient mice
Source: PLoS One. 2019 Aug 22;14(8):e0221528. doi: 10.1371/journal.pone.0221528 (PMC6705798; doi:10.1371/journal.pone.0221528)
Supplement: S1 Table — (DOCX) [file pone.0221528.s005.docx]

**S1 Table. List of antibodies used in this study**

| **Antigen** | **Clone** | **Supplier** |
| --- | --- | --- |
| CD115 | AFS98 | eBioscience/Thermo Fisher |
| CD11b | M1/70 | eBioscience/Thermo Fisher |
| CD11c | N418 | eBioscience/Thermo Fisher |
| CD135 | A2F10 | eBioscience/Thermo Fisher |
| CD16/32 | 93 | eBioscience/Thermo Fisher |
| CD19 | 6D5 | eBioscience/Thermo Fisher |
| CD25 | PC-61.5 | eBioscience/Thermo Fisher |
| CD34 | RAM34 | BDBiosciences |
| CD3ε | 145-2C11 | eBioscience/Thermo Fisher |
| CD4 | RM4-5 | eBioscience/Thermo Fisher |
| CD45 | 30-F11 | BDBiosciences |
| CD45R | RA3-6B2 | eBioscience/Thermo Fisher |
| CD8 | 53-6.7 | eBioscience/Thermo Fisher |
| CD86 | GL1 | eBioscience/Thermo Fisher |
| cKit | 2B8 | eBioscience/Thermo Fisher |
| F4/80 | T7 | eBioscience/Thermo Fisher |
| FoxP3 | FJK-16s | eBioscience/Thermo Fisher |
| Ly6C | HK1.4 | eBioscience/Thermo Fisher |
| Ly6G | 1A8-Ly6G | eBioscience/Thermo Fisher |
| Ly6G and Ly6C | RB6-8C5 | BDBiosciences |
| Ly-76 | TER-119 | BDBiosciences |
| MHCII | M5/114.5.2 | eBioscience/Thermo Fisher |
| NK1.1 | PK136 | BDBiosciences |
| S100A8 | 335806 | R&D Systems |
| S100A9 | 2A5 | In house |
| Sca1 | D7 | eBioscience/Thermo Fisher |
| TCRαβ | H57-597 | eBioscience/Thermo Fisher |
