# Supplementary material for: Enhanced myelopoiesis and aggravated arthritis in S100a8-deficient mice
Source: PLoS One. 2019 Aug 22;14(8):e0221528. doi: 10.1371/journal.pone.0221528 (PMC6705798; doi:10.1371/journal.pone.0221528)
Supplement: S2 Table — (DOCX) [file pone.0221528.s006.docx]

**S2 Table. Demographic and clinical data of the research project participants**

|  | **Controls** | **Rheumatoid arthritis** |
| --- | --- | --- |
| Age, years | 50 ± 8 | 55 ± 13 |
| Disease duration, months |  | 6.5 ± 2.5 |
| BMI | 27.6 ± 5.1 | 28.3 ± 8.4 |
| Female | 7 (70) | 10 (56) |
| Smoker | 0 (0) | 6 (32) |
| Rheumatoid Factor | 0 (0) | 14 (74) |
| Anti-CCP | 0 (0) | 14 (74) |
| CDAI |  | 41.7 ± 15.3 |
| DAS28-CRP |  | 5.5 ± 1.1 |
| NSAIDs | 0 (0) | 8 (42) |
| Anti-malarial | 0 (0) | 6 (32) |

Values presented as mean ± standard deviation or frequency (percentage). BMI: body mass index, Anti-CCP: anti-cyclic citrunillated peptide, CDAI: clinical disease activity index, DAS28-CRP: Disease Activity Score 28-joint count C reactive protein, NSAIDs: Nonsteroidal Anti-inflammatory Drugs
